# Maternal and infant serum ferritin concentrations across pregnancy and postpartum: a longitudinal study in Norway

**DOI:** 10.29219/fnr.v70.14217

**Published:** 2026-06-23

**Authors:** Synnøve Næss Sleire, Inger Aakre, Lisbeth Dahl, Tonje Eiane Aarsland, Rebekka Sandvik, Marian Kjellevold, Maria Wik Markhus

**Affiliations:** 1 Department of Seafood, Nutrition and Environmental State, Institute of Marine Research, Bergen, Norway; 2 Women’s Clinic at Lillehammer Hospital, Innlandet Hospital Trust, Lillehammer, Norway; 3 Department of Clinical Medicine, University of Bergen, Bergen, Norway

**Keywords:** iron status, ferritin, pregnancy, infants, breastfeeding, Norway

## Abstract

**Introduction:**

Adequate iron status is particularly important during pregnancy and infancy, as iron deficiency can pose health risks to the woman, developing fetus, and future child. However, data in these population groups are scarce in Norway.

**Aim:**

To describe serum ferritin concentrations in a cohort of pregnant women in Norway, followed through the postpartum period, and their infants. Further, to examine associations between maternal and infant serum ferritin concentrations, and to assess the influence of dietary supplement use and breastfeeding status on serum ferritin levels.

**Methods:**

In this longitudinal cohort study, 137 pregnant women in Norway were enrolled and followed at gestational week 18 and 36, and at 3 and 6 months postpartum together with their infants. Infant and maternal serum ferritin concentrations were measured.

**Results:**

At gestational week 18, 14% of pregnant women were iron deficient (serum ferritin < 15 µg/L), and 44% had depleted iron stores (< 30 µg/L). By gestational week 36, the prevalence of iron deficiency had increased to 65% and depleted stores to 95%. Infant ferritin concentrations declined between 3 and 6 months of age. At 3 months, 96% of infants had sufficient iron status (≥ 50 µg/L). At 6 months, 90% remained sufficient, while 10% were iron deficient (< 15 µg/L). No associations were observed between iron status and dietary iron supplement use or breastfeeding status.

**Conclusion:**

A substantial proportion of the women were found to have iron deficiency, and depleted iron stores both during pregnancy and the postpartum period measured by serum ferritin concentrations. In contrast, the infants generally showed adequate iron status, and although serum ferritin levels declined between 3 and 6 months of age, the prevalence of iron deficiency remained low.

## Popular scientific summary

- Low iron levels were common among pregnant and postpartum women in Norway and became increasingly prevalent as pregnancy progressed.
- Most infants had sufficient iron levels during their first six months of life, even though their iron stores gradually declined over time.
- We found no clear link between iron supplement use or breastfeeding and iron levels in either the mothers or their infants.

Adequate iron status is particularly important in pregnancy and infancy, as iron deficiency can pose health risks to the woman, developing fetus, and future child (1). Iron deficiency in pregnancy and early childhood increases the risk of low birth weight and preterm birth (2), as well as reduced child development and growth during the first years of life (3). Iron requirements are especially high during these periods due to the demands of rapid growth and development (4, 5).

Globally, it is estimated that more than 4 billion people do not consume enough iron (6), and iron deficiency is the leading cause of anemia, affecting almost 2 billion people globally (7). Pregnant women and children are particularly vulnerable, and the World Health Organization (WHO) estimates that 37% of pregnant women and 40% of children below 5 years of age are affected (8). Although the prevalence is lower in Europe, anemia still affects 24.3% of pregnant women (9), and iron deficiency (defined as low iron stores) affects 28–85% of pregnant women depending on supplementation use (10).

Exclusive breastfeeding is recommended for infants during the first 6 months (9, 11); however, human breast milk is naturally low in iron (0.2–0.4 mg/L) (12). Therefore, infants rely on the iron stores accumulated prenatally to meet their needs during this period (13). By 6 months of age, infant iron stores deplete, and breastmilk alone is no longer sufficient to meet iron requirements. At this stage, dietary iron from complementary foods is needed (14). However, infants at risk of iron depletion may benefit from iron-rich complementary foods introduced before 6 months of age (15).

There is a lack of recent data on iron status in pregnant and postpartum women, as well as infants, in Norway and other European countries. A study from The Norwegian Mother, Father and Child Cohort Study (MoBa) (*n* = 2,990) conducted between 2002 and 2008 showed that a substantial number of women had low iron stores in pregnancy (44% had low or depleted iron stores indicated by serum ferritin < 30 µg/L) (16). Furthermore, a study of postpartum women in Norway (*n* = 573), conducted between 2008 and 2010, showed a high prevalence of iron deficiency (39% had depleted iron stores indicated by serum ferritin < 15 µg/L) (17). Also, few studies have included both data on pregnant women and followed them with their infants in the first year of life. Thus, there is a need for updated data on iron status in these population groups in Norway.

## Objective

The aim of this study is to describe serum ferritin concentrations in a cohort of pregnant women in Norway, followed into the postpartum period, and in their infants. A further aim is to examine associations between maternal and infant serum ferritin concentrations, and to assess the influence of dietary supplement use and breastfeeding status on serum ferritin levels.

## Methods

### Study design and participants

This study constitutes a secondary analysis of data derived from the ‘Mommy’s Food’ study (NCT02610959), which involved the enrollment of pregnant women and subsequent follow-up with their infants postpartum in Bergen, Norway, between 2016 and 2018. The study was originally a two-armed randomized controlled trial where the primary and secondary aims were to investigate if an increased intake of lean fish (Atlantic cod) in pregnancy (gestational week 20–36) had an impact on I) maternal iodine status and II) infant development. Detailed descriptions of the study design, protocol, and results from the primary and secondary aims are available elsewhere (18, 19). There were no differences in serum ferritin concentration between the intervention and control group at any time-point (20). Therefore, in the current article, we wanted to explore ferritin concentrations in a longitudinal study design. Thus, participants from both the intervention and the control arms of the study were included in a secondary analysis of the data.

The current longitudinal cohort comprised 137 pregnant women, who were recruited at 18 weeks of gestation and monitored at 36 weeks of gestation, as well as at 3 and 6 months postpartum, along with their infants. Recruitment occurred at the Women’s Clinic at Haukeland University Hospital, located in Health Region West of Norway, between January 2016 and February 2017. In addition, online broadcast was used for recruitment to achieve the required participation rate. Inclusion criteria were first-time pregnant women, singleton pregnancy, gestational week ≤ 19, and Norwegian speaking and/or able to understand Norwegian writing (owing to questionnaires in Norwegian).

**Fig. 1:**
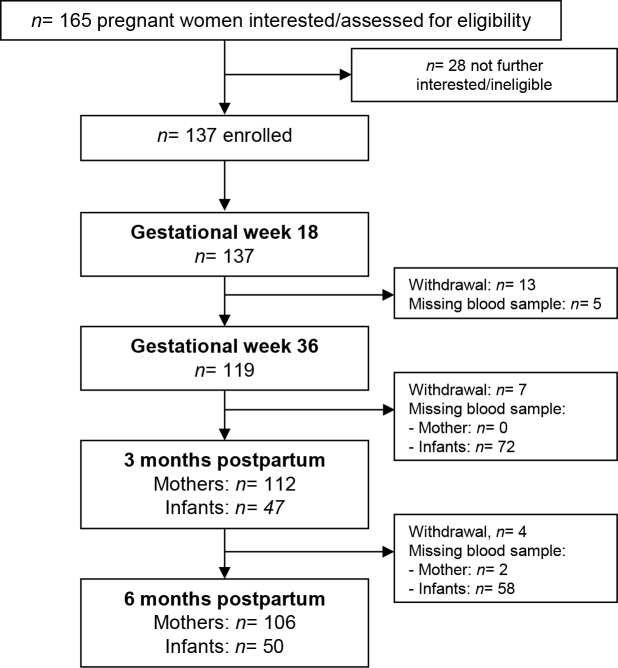
Flow chart of the participants throughout the study.

During study visits at gestational week 18 and 36, and at 3 and 6 months postpartum, blood samples were collected, and questionnaires were administered to gather data on demographics, anthropometry, dietary intake, and breastfeeding status. Due to participant attrition, difficulties in obtaining blood samples from infants, and prioritization of whole blood before serum samples, some data points are missing. All participants with available data for the relevant outcomes were included to maximize the sample size and reduce the impact of missing data. Figure 1 illustrates the study population flow and the availability of data at each time point.

### Infant and maternal ferritin concentrations

For analyses of ferritin concentrations, blood samples were obtained from both mothers and infants during pregnancy (at 18 and 36 weeks of gestation) and at 3 and 6 months postpartum by a trained phlebotomist. Venous blood samples for serum preparation were collected using BD Vacutainer^®^ SST™ vials II Advanced (Becton, Dickinson and Co.; NJ, US) and allowed to coagulate for at least 30 min before centrifugation (1,000–3,000 × g, room temperature, 10 min) within 60 min post-venepuncture. Following separation, serum samples were stored at −80°C for up to 3 months, pending analysis. In cases where venepuncture was not feasible in infants, capillary blood was collected from the heel or fingertip (depending on age and/or body weight) using BD Microtainer^®^ Blood Collection Tubes (Becton, Dickinson and Co.; Franklin Lakes, NJ, US). Heel pricks were performed using a Tenderfoot ITC^®^ heel-incision device (Accriva Diagnostics; San Diego, CA, US), and finger pricks were conducted with ACCU-CHEK^®^ Safe-T-Pro Plus lancets (Roche Diagnostics; Rotkreuz, Switzerland). To facilitate capillary blood sampling, the heel or finger was pre-warmed with a hot water balloon to promote vasodilation and ensure adequate blood flow. Infants were administered 12 mL of a 25% sucrose-water solution prior to blood sampling to mitigate pain-related stress.

Serum ferritin concentrations were further analyzed at Fürst Medical Laboratory in Oslo, Norway, by an immunoturbidimetric method using an Advia Chemistry XPT system (Siemens Medical Solutions Diagnostica). The coefficient of variation (CV) for serum ferritin concentration was 2.5%.

To assess the prevalence of iron deficiency and low iron stores, different cut-off values of ferritin concentrations were used. For the mothers, iron deficiency was defined as serum ferritin concentration < 15 µg/L, both during pregnancy and post-partum, consistent with the guidelines by the Nordic Nutrition Recommendations 2023 (21), the WHO (22) and the laboratory (23). Further, depleted iron stores were defined as serum ferritin < 30 µg/L (10, 16). Maternal iron status in pregnancy was also compared against the cut-off < 70 µg/L, as this is the cut-off used for recommendations of iron supplementation in pregnancy by the Norwegian Directorate of Health (24). For the infants, the cut-off used to define iron deficiency was < 50 µg/L at 3 months of age and < 15 µg/L at 6 months of age (23).

### Breastfeeding status

Breastfeeding status at 3 and 6 months postpartum was assessed using a 24-h dietary recall during study visits. The purpose of this variable was to describe the type of infant feeding; breast milk, formula, or both, rather than to distinguish between exclusive and predominant breastfeeding. Based on reported intake, infants were categorized as: *breastfed* (receiving breast milk and no formula), *mixed-milk-fed* (receiving both breast milk and formula), or *formula-fed* (receiving only formula and no breast milk).

At 3 months, all infants who were breastfed were exclusively breastfed according to the WHO definition (25), and at 6 months only 3% were exclusively breastfed. Thus, infants in the breastfeeding category at 6 months could also be receiving complementary foods.

For statistical analyses, the mixed-milk-fed and formula-fed groups were combined into a single category, *mixed-milk-fed or formula-fed*, due to the low number of participants in these groups.

### Dietary iron supplement use

Data regarding maternal and infant iron supplement use were collected through questionnaires completed by mothers at 3 and 6 months postpartum. This included a food frequency questionnaire (FFQ) with the recall period aimed to be the last 3 months of the participant’s habitual diet. The FFQ included questions regarding the use of multivitamin-mineral (including iron) and pure iron supplements. Supplement users included the frequency responses ‘1–3 times weekly’, ‘4–6 times weekly’, and ‘daily’. Non-supplement users included the frequency responses ‘never’ or ‘1–3 times monthly’.

### Background variables

Maternal and infant background characteristics were collected via electronic questionnaires administered at recruitment (18 weeks of gestation) and at 3 and 6 months postpartum. The questionnaires gathered data on maternal age, education level, pre-pregnancy weight and height, nicotine use (smoking and snuff), and gestational age at birth. Additionally, information on infant sex, birth weight, current weight and length, age at introduction of complementary foods, and specific ages for anthropometric measurements was included. Premature birth was defined as occurring before 37 weeks gestation, and low birth weight was defined as less than 2,500 grams. Infant weight-for-age *z*-scores at ages 3 and 6 months were calculated using the WHO child growth standard and the WHO Anthro Packages (26). Missing anthropometric data from the questionnaires were collected at the study visits at 3 and 6 months.

### Ethics

Ethical approval for this study was granted by the Norwegian Regional Committees for Medical and Health Research Ethics West (reference REK 2015/879). The study is registered at ClinicalTrials.gov (NCT02610959) as of 15 November 2015. The study was conducted in accordance with the ethical principles outlined in the latest version of the Declaration of Helsinki. Written informed consent was obtained from all participating pregnant women after they received both written and oral information about the study. Participation was voluntary, and participants were informed that they could withdraw from the study at any time without providing a reason, as emphasized in the informed consent declaration. The provision of biological samples was optional, and mothers provided verbal consent for infant blood sampling at each study visit.

### Statistical analyses

Variables were tested for normality by visual inspection of Q-Q plots and histograms. Descriptive results are reported as proportions (%) for categorical variables. For continuous variables, means ± standard deviations (SDs), medians, or percentiles are reported as appropriate. Correlations between maternal and infant ferritin concentrations were assessed using Spearman’s rank order correlation coefficient (Spearman’s rho). Effects of maternal iron supplementation on maternal ferritin concentrations and the effects of breastfeeding status on infant ferritin concentrations were tested with generalized linear models. The adjusted models included adjustments for maternal age, pre-pregnancy BMI, educational level, and nicotine use in pregnancy. Statistical analyses were performed using Statistical Package for the Social Sciences (SPSS) for Windows, version 27 (International Business Machines [IBM] Corporation).

## Results

### Characteristics of study population

Figure 1 illustrates the flow chart of the study population. Initially, 165 pregnant women expressed interest, with 137 enrolling and attending the first follow-up at gestational week 18. Participation decreased to 119 at week 36, 112 at 3 months postpartum, and 106 at 6 months postpartum. Due to missing blood samples, 47 and 50 infants were included at 3 and 6 months, respectively.

Table 1 details maternal and infant characteristics. At recruitment, the mean maternal age was 29.3 years, with 86% holding a university/college degree. The mean gestational age at birth was 40 weeks, with 5% born prematurely (< 37 weeks). The mean (SD) birth weight was 3,517 (588) g, with 4.5% classified as low birth weight (< 2,500 g). At 3 months, 79% of infants were exclusively breastfed, 15% mixed-milk-fed, and 6% formula-fed. At 6 months, 74% were breastfed (none were exclusively breastfed), 22% mixed-milk-fed, and 4% formula-fed. The mean (SD) age for introducing complementary foods was 20 (4) weeks.

**Table 1:** Maternal and infant characteristics

Characteristic	*N*	Value
**Maternal** ^a^		
Age (years), mean (SD)	135	29.3 (3.8)
Pre-pregnancy BMI (kg/m^2^), mean (SD)	132	23.1 (4.0)
Education level, *n* (%)	133	
Elementary school		2 (1.5)
High school		17 (13)
≤ 4 years university/college		33 (25)
> 4 years university/college		81 (61)
Iron supplement users^b^		
GW 18	124	
Yes, *n* (%)		60 (48)
No, *n* (%)		64 (52)
GW 36	104	
Yes, *n* (%)		67 (64)
No, *n* (%)		37 (36)
3 M PP	90	
Yes, *n* (%)		49 (54)
No, *n* (%)		41 (46)
6 M PP	73	
Yes, *n* (%)		15 (21)
No, *n* (%)		58 (79)
**Infant** ^c^	**N**	
Age at study visit (months)		
3 M, mean (SD)	47	3.4 (0.3)
6 M, mean (SD)	50	6.3 (0.3)
Sex, *n* (%) girls	62	31 (50)
Gestational week at birth, mean (SD)	62	40 (1.6)
Premature (< GW 37), *n* (%)		4 (6.5)
Birth weight (g), mean (SD)	44	3,517 (588)
Low birth weight (< 2,500 g), *n* (%)		2 (4.5)
Weight-for-age *z*-score		
3 M, mean (SD)	43	0.15 (0.91)
3 M, < 2 SD (underweight), *n* (%)	43	0
6 M, mean (SD)	41	0.59 (1.0)
6 M, < 2 SD (underweight), *n* (%)	41	0
Breastfeeding status^d^	62	
3 M	47	
Breastfed, *n* (%)		37 (79)
Mixed-milk-fed, *n* (%)		7 (15)
Formula-fed, *n* (%)		3 (6)
6 M	49	
Breastfed, *n* (%)		36 (74)
Mixed-milk-fed, *n* (%)		11 (22)
Formula-fed, *n* (%)		2 (4)
Age (weeks) at introduction of complementary foods, mean (SD)	38	20.3 (4.1)

BMI: body mass index; IQR: interquartile range; GW: gestational week; M: months; PP: postpartum; SD: standard deviation.

a Maternal characteristic retrieved from an electron questionnaire completed at gestational week 18.

b Iron supplement user include iron supplements from multimineral and pure iron supplements. Supplement users were defined as use more than weekly (1–3 times/week or more).

c Infant characteristics retrieved from an electron questionnaire completed among the mothers at 3 and 6 months postpartum.

d Includes the categories ‘breastfed’ (no use of formula) ‘mixed-milk-fed’ (breastfed and formula-fed), and ‘formula-fed’ (only use of formula, no breastfeeding).

Iron supplement use was reported by 48% of women at week 18, 64% at week 36, 54% at 3 months postpartum, and 21% at 6 months postpartum, including both multimineral tablets and pure iron supplements. Pure iron supplement use was 7, 24, 19, and 12% at these respective time points. Among infants, only one at both 3 and 6 months used dietary iron supplements.

### Maternal and infant ferritin concentrations

**Fig. 2:**
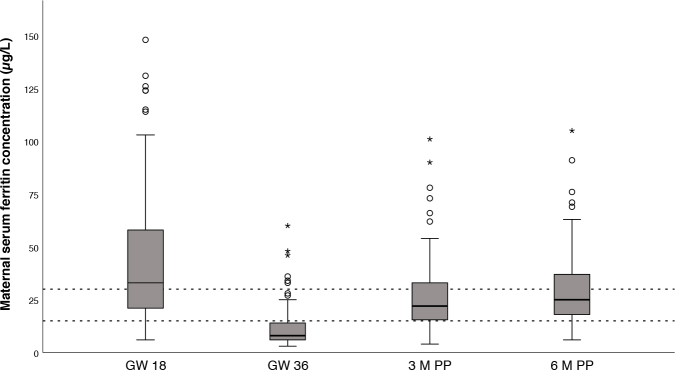
Maternal serum ferritin concentrations in pregnancy (gestational week 18 and 36) and post-partum (3 and 6 months). The boxes indicate the upper (75th percentile) and lower (25th percentile) quartile with the thick black line as the median (50th percentile). The dotted lines show serum ferritin = 30 and 15 µg/L. GW: gestational week; M: months; PP: postpartum.

**Fig. 3:**
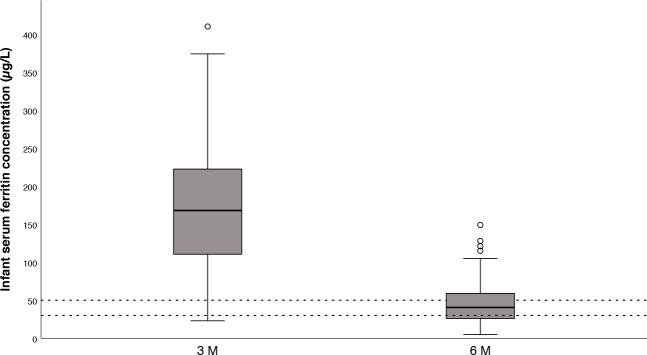
Infant serum ferritin concentrations at ages 3 and 6 months. The boxes indicate the upper (75th percentile) and lower (25th percentile) quartile with the thick black line as the median (50th percentile). The dotted lines show serum ferritin = 50 and 30 µg/L. M: months.

Table 2 and Figs. 2 and 3 present maternal and infant serum ferritin concentrations and their distribution across different cut-offs. Maternal serum ferritin decreased from gestational week 18 to 36 and increased postpartum. At gestational week 18, 14% of women were iron deficient (< 15 µg/L), 44% had depleted iron stores (< 30 µg/L), and 80% had < 70 µg/L, the cut-off for iron supplementation in Norway. By week 36, iron deficiency increased to 65% and depleted stores to 95%. At 3 months postpartum, 22% were iron deficient and 67% had depleted stores, with similar figures at 6 months postpartum. Infant ferritin concentrations decreased from 3 to 6 months, with 96% having sufficient iron status (≥ 50 µg/L) at 3 months, and 90% at 6 months, with 10% iron deficient (< 15 µg/L).

**Table 2:** Maternal and infant iron status measured by serum ferritin concentrations

Serum ferritin concentrations (µg/L)^a^							
**Mothers**	***n***	**Mean (SD)**	**Median (IQR)**	**< 15, *n* (%)**	**≥ 15 to < 30, *n* (%)**	**≥ 30 to < 70, *n* (%)**	**≥ 70, *n* (%)**
GW 18	137	45 (37)	33 (21–58)	19 (14%)	41 (30%)	50 (36%)	27 (20%)
GW 36	119	12 (9)	8 (6–14)	93 (68%)	20 (17 %)	6 (5%)	0
3 M PP	112	26 (17)	22 (15–33)	25 (22%)	51 (46%)	32 (29%)	4 (4%)
6 M PP	106	29 (17)	25 (18–37)	15 (14%)	56 (53%)	31 (29%)	4 (4%)
**Infants**	***n***	**Mean (SD)**	**Median (IQR)**	**< 15, *n* (%)**	**≥ 15 to < 30, *n* (%)**	**≥ 30 to < 50, *n* (%)**	**≥ 50, *n* (%)**
3 M	47	169 (78)	168 (109–223)	0	1 (2%)	1 (2%)	45 (96%)
6 M	50	48 (32)	41 (26–59)	5 (10%)	12 (24%)	13 (26%)	20 (40%)

M: months; PP: postpartum; GW: gestational week.

a Mothers: serum ferritin < 15 µg/L defined as iron deficiency (21, 22); < 30 µg/L defined as depleted iron stores (10, 16); < 70 µg/L defined as cut-off used for recommendations of iron supplementation in pregnancy by the Norwegian Directorate of Health (24). Infants: cut-off used to define iron deficiency was < 50 µg/L at 3 months of age and < 15 µg/L at 6 months of age (23).

### Correlation between maternal and infant ferritin concentrations

Maternal ferritin concentrations were highly positively correlated across all time points (Table 3), particularly between week 18 and 36 (*r* = 0.435, *P* < 0.001), and between 3 and 6 months postpartum (*r* = 0.608, *P* < 0.001). Infant ferritin concentrations at 3 months were positively correlated with those at 6 months (Table 3). Infant ferritin concentrations were weakly negatively correlated with maternal ferritin concentrations, though only significantly at 3 months of age with maternal ferritin at week 36 (*r* = –0.345, *P* = 0.022).

**Table 3:** Correlation matrix of maternal and infant ferritin concentrations

		Mothers				Infants	
		Gestational week 18	Gestational week 36	3 months postpartum	6 months postpartum	3 months	6 months
Mothers	*n*						
GW 18	137	1.00					
GW 36	119	0.435 (*P* < 0.001)	1.00				
3 M PP	112	0.250 (*P* = 0.008)	0.372 (*P* < 0.001)	1.00			
6 M PP	106	0.348 (*P* < 0.001)	0.489 (*P* < 0.001)	0.608 (*P* < 0.001)	1.00		
Infants	*n*						
3 M	47	–0.217 (*P* = 0.143)	**–**0.345 (*P* = 0.022)	–0.232 (*P* = 0.117)	–0.236 (*P* = 0.123)	1.00	
6 M	50	–0.189 (*P* = 0.190)	–0.075 (*P* = 0.611)	–0.047 (*P* = 0.748)	–0.125 (*P* = 0.385)	0.647 (*P* < 0.001)	1.00

The results are presented as Spearman’s rho (*P*-value).

GW: gestational week; M: months; PP: postpartum.

### Effects of dietary iron supplement use and breastfeeding status on serum ferritin concentrations

Table 4 gives the effects of maternal iron supplementation on maternal ferritin concentrations. Maternal iron supplement users had higher ferritin concentrations at 3 months postpartum [mean (SD): 31 (20) µg/L] compared to non-users [mean (SD): 22 (14) µg/L] (adjusted *P* = 0.016). No significant differences were observed at other time points. No clear differences were found in infant ferritin concentrations between breastfed, mixed-fed, or formula-fed infants at 3 or 6 months of age (Table 5).

**Table 4:** Effects of maternal iron supplementation on maternal ferritin concentrations

Time-point	Ferritin concentrations (µg/L)				Effects of maternal iron supplement use on ferritin concentrations (non-users vs. users)			
	Iron supplement user^a^ (reference)		Non-supplement user		Unadjusted^b^		Adjusted^c^	
	*N*	Mean (SD)	*N*	Mean (SD)	β (95% CI)	*P*	β (95% CI)	*P*
**Mothers**								
GW 18	60	45 (35)	64	47 (42)	2.12 (–11.7, 15.9)	0.761	1.24 (–13.1, 15.6)	0.864
GW 36	67	12 (8.1)	37	9.8 (10)	–1.94 (–5.56, 1.69)	0.291	–1.63 (–5.36, 2.11)	0.390
3 M	49	31 (20)	41	22 (14)	–8.80 (–16.3, –1.26)	0.023	–9.45 (–17.1, –1.82)	0.016
6 M	26	28 (14)	47	28 (15)	–0.84 (–8.03, 6.34)	0.815	–2.45 (–9.78, 4.88)	0.507

BMI: body mass index; CI: confidence interval; SD: standard deviation.

a Iron supplement user include iron supplements from multimineral and pure iron supplements. Supplement users were defined as more than weekly (1–3 times/week or more) use.

b Generalized Linear Models. Iron supplement user (reference category) vs. non-supplement user.

c Generalized Linear Models adjusted for age, pre-pregnancy BMI, educational level, and nicotine use in pregnancy. Iron supplement user (reference category) vs. non-supplement user.

**Table 5:** Effects of breastfeeding status on infant ferritin concentrations

Time-point	Ferritin concentrations (µg/L)				Effects of breastfeeding status on ferritin concentrations (mixed-milk-fed or formula-fed vs. breastfed)			
	Breastfed^a^ (reference)		Mixed-milk-fed or formula-fed		Unadjusted^b^		Adjusted^c^	
	*N*	Mean (SD)	*N*	Mean (SD)	β (95% CI)	*P*	β (95% CI)	*P*
3 M	37	166 (81.8)	10	180 (63.4)	13.8 (–42.5, 70.2)	0.622	15.8 (–43.6, 75.2)	0.594
6 M	36	49.1 (32.2)	13	43.5 (32.3)	–5.59 (–26.6, 15.4)	0.594	–2.43 (–26.7, 21.9)	0.841

BMI: body mass index; CI: confidence interval; SD: standard deviation.

a Breastfeeding status.

b Generalized Linear Models. Breastfed (reference category) vs. mixed-milk-fed or formula-fed.

c Generalized Linear Models adjusted for age, pre-pregnancy BMI, educational level, and nicotine use in pregnancy. Breastfed (reference category) vs. mixed-milk-fed or formula-fed.

## Discussion

In this longitudinal study of pregnant women followed postpartum with their infants, we present data on iron status assessed through serum ferritin concentrations. A substantial proportion of the women were found to have iron deficiency (ferritin < 15 µg/L), and depleted iron stores (ferritin < 30 µg/L) during both pregnancy and the postpartum period. In contrast, the infants generally demonstrated adequate iron status, and although serum ferritin levels declined between 3 and 6 months of age, the prevalence of iron deficiency among infants remained low.

In the present study, 14% of the women were iron deficient and 44% had depleted iron stores in gestational week 18. These findings are comparable to previous studies in Norway assessing iron status at similar gestational timepoints (16, 17), as well as to data from other European countries (10). The proportion of women with iron deficiency and depleted iron stores increased substantially over the course of pregnancy in our study, reaching 65 and 95%, respectively, by gestational week 36. It is well recognized that serum ferritin concentrations decline progressively during pregnancy due to physiological hemodilution, which limits its utility as a marker of iron status in late pregnancy. At this stage, hemoglobin levels may provide a better indication of the clinical impact of iron status. Nonetheless, serum ferritin and hemoglobin concentrations are closely correlated during pregnancy, and low ferritin levels are associated with an increased risk of low hemoglobin concentrations and iron deficiency anemia (16). Altogether, these findings suggest that the high prevalence of iron deficiency observed in our study likely reflects a true depletion of iron stores, which progress over the course of pregnancy. This is concerning, as iron deficiency during pregnancy has been associated with adverse maternal and fetal outcomes, including preterm birth, low birthweight, reduced child neurodevelopment, and generally reduced maternal health and wellbeing (27–29). Furthermore, even in the absence of overt anemia, low iron stores may contribute to mild but clinically relevant symptoms such as fatigue, reduced physical and cognitive function, and increased vulnerability during subsequent pregnancies (30). Nevertheless, the long-term implications of depleted iron stores among women in Norway remain unclear. Given the high prevalence of low ferritin in women of reproductive age in Norway, future research should examine whether sustained iron deficiency contributes to functional consequences such as reduced work capacity, cognitive effects, or increased vulnerability in later pregnancies. Addressing these knowledge gaps will be important for understanding the public health significance of the iron status patterns observed in this population.

One potential explanation for the high prevalence of iron deficiency observed in this study is the low use of iron supplementation during pregnancy. While routine iron supplementation in pregnancy is implemented in many countries worldwide, it is not currently recommended in Norway. Instead, national guidelines advise assessment of ferritin levels in the first trimester, with iron supplementation (40–60 mg/day) recommended for women with ferritin concentrations below 70 µg/L. In our study, 80% of the pregnant women had ferritin concentrations < 70 µg/L at gestational week 18 indicating a need for iron supplementation. However, only 48% of the women reported using iron supplements at this timepoint, and among them, the majority were taking a multivitamin-mineral supplement, with only 7% using iron-only supplements. Furthermore, the iron content in most multivitamin and prenatal supplements is typically around 15 mg/day, which is considerably lower than the recommended dosage for iron supplementation during pregnancy. This was also evident when we examined the effects of iron supplementation on iron status, as the impact of supplementation was generally limited, with the exception of a modest effect observed at 3 months postpartum. Previous studies have reported positive effects of iron supplementation on ferritin concentrations (31–33); therefore, it is likely that the iron dose used by participants in this study was too low to have a substantial impact on iron status.

Ferritin concentrations increased among the mothers after birth; however, 22 and 14% still had ferritin levels indicative of iron deficiency at 3 and 6 months postpartum, respectively, and 67 and 68% showed evidence of depleted iron stores at these time points. These proportions were somewhat lower compared to another study in Norway among 573 women at 14 weeks postpartum, which reported iron deficiency in 39% (17). Notably, that study included a multi-ethnic population and found that the prevalence of iron deficiency was significantly higher in certain minority groups, which may explain the higher overall prevalence observed. These findings highlight the importance of assessing iron status not only during pregnancy, but also in the postpartum period to ensure adequate iron stores among women. When examining the correlations of maternal serum ferritin levels across all time points (gestational week 18, gestational week 36, and at 3 and 6 months postpartum), we found a consistent and significant positive correlation. This suggests that adequate serum ferritin levels in early pregnancy may contribute to maintaining sufficient iron status later in pregnancy and into the postpartum period, emphasizing the importance of initiating strategies to support optimal iron status as early as possible in pregnancy.

During pregnancy, the fetus accumulates iron stores to support adequate iron status during the first 6 months of life, prior to the introduction of complementary feeding, as the iron content of breastmilk is generally low. Despite the high prevalence of iron deficiency observed among the pregnant women in our study, the infants did not appear to be affected of iron deficiency in early infancy. At 3 months of age, only 4% of infants had serum ferritin concentrations indicative of iron deficiency. This suggests that fetal iron accretion is prioritized during pregnancy, potentially at the expense of maternal iron stores. However, ferritin levels declined markedly from 3 to 6 months of age, and by 6 months, 10% of the infants were iron-deficient (serum ferritin < 15 µg/L). Even though declining ferritin concentrations is a physiological response to normal development as the fetal iron stores are used up, this finding underscores the importance of introducing iron-rich complementary foods from 6 months of age. In Norway, the prevalence of exclusive breastfeeding is high. Therefore, it is crucial that complementary foods introduced at around 6 months of age are rich in bioavailable iron to prevent further depletion of iron stores. The European Food Safety Authority (EFSA) has also stated that infants at risk of iron depletion may benefit from the introduction of iron-rich complementary foods before 6 months of age, and that there is no evidence that starting complementary feeding between 4 and 6 months has adverse effect (15).

Although breastmilk is inherently low in iron, it has been debated whether formula-fed infants maintain better iron status due to the higher iron content of infant formula. However, the bioavailability of iron in breast milk has been shown to be higher than in formula, which is important in addition to the absolute amount of iron ingested (34). In the present study, we found no significant difference in ferritin concentrations between breastfed and formula-fed infants. However, given the small number of formula-fed infants in our sample, these findings should be interpreted with caution.

Few studies have assessed infant iron status during the first year of life; nevertheless, the available data are generally consistent with our findings. A Norwegian study of infants aged 6–24 months reported a mean ferritin concentration of 57 µg/L at 6 months, with 6% of infants classified as iron-deficient (35). Notably, in the mentioned study, the prevalence of iron deficiency increased to 21% at 12 months and 29% at 24 months, highlighting the progressive risk of iron depletion with age (35). Another study from Norway of 252 infants aged 3–7 months reported a mean ferritin concentration of 74 µg/L, although 25% of the infants exhibited iron deficiency (36). In this study, they also found that ferritin concentrations < 51 µg/L were associated with suboptimal gross motor scores in infants 3–7 months (36). Though it should also be noted that the thresholds employed to define iron deficiency vary across studies, which complicates direct comparisons between studies.

### Strengths and limitations

A key strength of this study is the use of longitudinal data, with iron status measured at multiple time points in both mothers and infants. However, the study also has some limitations. The number of available serum samples from infants was limited due to the challenges of obtaining blood samples in this age group, which reduced the sample size and may have constrained the statistical power to detect small to moderate associations. In addition, missing data at several time points further reduced the number of complete cases available for longitudinal analyses, and results should therefore be interpreted with caution. In addition, apart from ferritin concentrations, we did not assess other biomarkers of iron status, such as hemoglobin or soluble transferrin receptors, nor inflammatory marker, which would have provided a more comprehensive evaluation of iron status. Furthermore, as the study was not originally designed to specifically investigate iron status, detailed data on dietary iron intake or sources were not collected, which would have strengthened the interpretation of the findings.

## Conclusion

This study presents data on iron status, assessed by serum ferritin concentrations, in a small cohort of pregnant and postpartum women and their infants from the western part of Norway. A substantial proportion of women exhibited iron deficiency and depleted iron stores during both pregnancy and the postpartum period. In contrast, most infants maintained adequate iron status, with a low prevalence of iron deficiency despite a decline in serum ferritin levels between 3 and 6 months of age. No associations were observed between iron status and dietary iron supplement use or breastfeeding status.
